# Immunomodulatory effect of Hawthorn extract in an experimental stroke model

**DOI:** 10.1186/1742-2094-7-97

**Published:** 2010-12-30

**Authors:** Chinnasamy Elango, Sivasithambaram Niranjali Devaraj

**Affiliations:** 1 Department of Biochemistry, University of Madras, Guindy Campus, Chennai-600 025, Tamil Nadu, India

## Abstract

**Background:**

Recently, we reported a neuroprotective effect for Hawthorn (*Crataegus oxyacantha*) ethanolic extract in middle cerebral artery occlusion-(MCAO) induced stroke in rats. The present study sheds more light on the extract's mechanism of neuroprotection, especially its immunomodulatory effect.

**Methods:**

After 15 days of treatment with Hawthorn extract [100 mg/kg, pretreatment (oral)], male Sprague Dawley rats underwent transient MCAO for 75 mins followed by reperfusion (either 3 or 24 hrs). We measured pro-inflammatory cytokines (IL-1β, TNF-α, IL-6), ICAM-1, IL-10 and pSTAT-3 expression in the brain by appropriate methods. We also looked at the cytotoxic T cell sub-population among leukocytes (FACS) and inflammatory cell activation and recruitment in brain (using a myeloperoxidase activity assay) after ischemia and reperfusion (I/R). Apoptosis (TUNEL), and Bcl-xL- and Foxp3- (T_reg _marker) positive cells in the ipsilateral hemisphere of the brain were analyzed separately using immunofluorescence.

**Results:**

Our results indicate that occlusion followed by 3 hrs of reperfusion increased pro-inflammatory cytokine and ICAM-1 gene expressions in the ipsilateral hemisphere, and that Hawthorn pre-treatment significantly (*p *≤ 0.01) lowered these levels. Furthermore, such pre-treatment was able to increase IL-10 levels and Foxp3-positive cells in brain after 24 hrs of reperfusion. The increase in cytotoxic T cell population in vehicle rats after 24 hrs of reperfusion was decreased by at least 40% with Hawthorn pretreatment. In addition, there was a decrease in inflammatory cell activation and infiltration in pretreated brain. Hawthorn pretreatment elevated pSTAT-3 levels in brain after I/R. We also observed an increase in Bcl-xL-positive cells, which in turn may have influenced the reduction in TUNEL-positive cells compared to vehicle-treated brain.

**Conclusions:**

In summary, Hawthorn extract helped alleviate pro-inflammatory immune responses associated with I/R-induced injury, boosted IL-10 levels, and increased Foxp3-positive T_regs _in the brain, which may have aided in suppression of activated inflammatory cells. Such treatment also minimizes apoptotic cell death by influencing STAT-3 phosphorylation and Bcl-xL expression in the brain. Taken together, the immunomodulatory effect of Hawthorn extract may play a critical role in the neuroprotection observed in this MCAO-induced stroke model.

## Background

*Crataegus oxycantha *(Hawthorn) extract is used for the treatment of heart diseases all over the world. Its beneficial effects have been attributed to the presence of Oligomeric proanthocyanidins (OPCs) [1]. Our group has recently shown that an alcoholic extract of Hawthorn is able to protect the brain from ischemia/reperfusion injury in a rat stroke model [2]. Worldwide, stroke is the third leading cause of death and the most frequent cause of long term disability in adults [3]. The immune system plays a vital role during cerebral stroke and has always been a key area of interest for scientists. There is mounting evidence that inflammatory mechanisms of the immune system are activated within hours after cerebral ischemia, representing a key target for recent translational cerebrovascular research [4]. Researchers have also shown that animals subjected to a systemic inflammatory insult at the time of stroke are predisposed to develop an autoimmune response against brain tissue, which may be associated with worsened outcome [5].

Damage due to cerebral stroke is not limited to brain. Stroke has more systemic effects, in that it results in a spike in inflammatory cytokines during the initial activation phase which is followed by severe immunosuppression, linked to atrophy of the spleen and thymus in MCAO-induced stroke models in mice [6]. Several other studies have linked acute stroke to fatal infections. Vogelgesang et al [7] showed that loss of CD4^+ ^T cell activation in stroke patients contributes to stroke-induced immunosuppression. Chamorro et al [8], in a clinical study, showed that acute ischemic stroke is associated with an early activation of the sympathetic adrenomedullary pathway that lowers the threshold for infection and increases the risk of death.

Several studies have reported on the immunoregulatory properties of procyanidins, Miyake et al [9] showed that OPCs from Jatoba extract exert strong inhibitory effects in an experimental autoimmune encephalitis mouse model. Rutin and Chorogenic acid, two polyphenolic antioxidants present in smokeless tobacco extract, reportedly augment inducible cytokine messages, like IL-10, and inhibit IgE-mediated mast cell activation [10]. Pentamer to hectamer procyanidin fractions from Cocoa cause more than 50% inhibition in IL-2 gene expression in phytohemagglutinin-induced human peripheral blood mononuclear cells [11].

The immunomodulatory role of Hawthorn extract (OPC rich) has not been explored. With herbal supplements gaining importance in recent therapeutic strategies, delineating the mechanisms of action for herbal supplements already used as therapies may increase their potential uses against different human pathologies. Previously we have shown that Hawthorn extract pretreatment is able to reduce infarct volume and improve neurological function in MCAO-induced strokes in rats, and we emphasized the extracts' antioxidant properties in that article. Our present goal was to investigate a potential role for Hawthorn extract in regulating inflammatory cascades associated with ischemia/reperfusion-induced injury.

## Methods

### Chemicals and reagents

All reagents and chemicals used in this study were of analytical grade and were purchased from Sigma-Aldrich (St Louis, MO, USA) unless otherwise mentioned. All antibodies and isotype controls for FACS analysis were obtained from BD Biosciences (San Jose, CA).

### Animals

The International Animal Care and Use Committee (IACUC) approved the animal protocol used in this study. Male Sprague-Dawley rats (Harlan Laboratories Livermore CA, USA) weighing 300-320 g were used. Animals were allowed free access to food and water and were housed at 20°C.

### Alcoholic extract of Hawthorn tablets

Each heart care Hawthorn Extract tablet (Nature's Way Products. Incs. Springville, Utah) contains 80 mg of Hawthorn extract. The tablets were ground to a fine powder and absolute ethanol was added. This mixture was allowed to stand overnight at room temperature. The extract was centrifuged and filtered. This extract was air dried for 1-2 hours to allow the ethanol to evaporate. PBS was added to a final extract concentration of 100 mg/ml (H-100) of solution.

### Treatment groups

Randomly selected animals were used to represent the following three groups: (A) Sham: used as operated controls with no treatment, (B) Vehicle control: ischemia for 75 mins followed by 3 or 24 hr of reperfusion and (C) H-100: Pretreatment (oral, gavage) with Hawthorn extract 100 mg/kg for 15 days followed by ischemia for 75 mins followed by 3 or 24 hr of reperfusion. The vehicle and sham groups received the same volume of saline every day for 15 days.

### Surgical procedure

Transient focal cerebral ischemia was induced in rats by subjecting them to MCAO as described previously [12] with slight modification. Briefly, the rats were initially anesthetized with 4% isoflurane in 20% oxygen, and maintained at 2% isoflurane in oxygen during the procedure. Internal body temperature was maintained using external heating pads during surgery. Under a dissecting microscope, the right common carotid artery was first exposed and the occipital branch of the external carotid artery (ECA) was coagulated by electrocautery. A silicon-coated 4.0 monofilament nylon suture (Doccol Corporation, Redland CA) was used for better consistency. After an opening was made on the ECA, the suture was inserted through the ECA and into the internal carotid artery for a distance of 20 mm (in our hands inserting the suture for 20 mm gave us consistent infarcts for rats weighing 300-320 gm). The animals were maintained in this condition for 75 mins of ischemia. After 75 mins, the suture was removed and normal blood flow was restored. Animals were then placed in their home cages to recover from anesthesia.

### Measurement of myeloperoxidase (MPO) activity in brain

Brain cerebral cortical tissue obtained after 24 hrs of reperfusion was used to analyze myeloperoxidase (MPO) activity according to methods previously described by Liao et al [13], with slight modifications. Briefly, 50 ul of protein extract (Total protein concentration was 1.6 mg/ml for all groups), was added to each well of an ELISA plate followed by 250 μl of assay buffer (26.9 ml of water, 3 ml of 0.1 M sodium phosphate buffer (pH 7.0), 100 μl of 0.1 M hydrogen peroxide and Guaiacol 50 μl). These were mixed and the kinetics of the reaction was followed for 1 min with a spectrophotometer at 470 nm.

### Quantification of IL-10 in brain samples

We isolated brain tissue from rats following 75 mins of occlusion and 24 hrs of reperfusion. The tissue samples were homogenized and IL-10 ELISA was performed using a kit from BD Biosciences (San Jose, CA). The manufacturer's protocol was followed to analyze IL-10 concentration in brain homogenates. IL-10 levels were expressed as picograms/0.1 mg of total protein.

### RNA isolation and reverse transcription

RNeasy Mini kit from Qiagen (Valencia, CA) was used for the isolation of RNA from brain tissue. Total RNA was extracted from the brain according to the manufacturer's protocol. We used a Quantitect kit from Qiagen (Valencia, CA) for genomic DNA removal and reverse transcription.

### TaqMan real-time PCR in brain tissue

Relative real-time RT-PCR analysis of IL-1β, TNF-α, IL-6, ICAM-1 and rpL32 (as a house keeping gene) were performed using a protocol previously described by Berti et al, [14], with slight modifications. The primers and probes (Applied Biosystems, Foster City, CA) used in this study are reported in Table 1. 6-Carboxyflurescin (FAM) was used as the reporter dye; 6-carboxy-tetramethyl-rhodamine (TAMRA) was the quencher dye. Real-time PCR was performed using TaqMan Universal PCR master Mix (Applied Biosystems, Foster City, CA). A 20 X stock solution of primers (18 μM) and probes (5 μM) was prepared separately for each gene. Two-to-five nanograms of cDNA were used in each reaction. Amplification conditions included 2 mins at 50°C and 10 mins at 95°C, followed by a run for 40 cycles at 95°C for 15 seconds and 60°C for 1 min on 7900 HT sequence detection system, ABI Prism (Applied Biosystems, Foster City, CA).

**Table 1:** Details of primers (forward & reverse) and probes for different genes used for real -time PCR analysis.

Gene	Primer/Probe	Sequences
**IL-1β**	Primer (forward)	5'-CAC CTC TCA AGC AGA GCA CAG-3'
	Primer (reverse)	5'-GGG TTC CAT GGT GAA GTC AAC-3'
	Probe (antisense)	5'-TGT CCC GAC CAT TGC TGT TTC CTA GG-3'

**TNF-α**	Primer (forward)	5'-CCA GGA GAA AGT CAG CCT CCT-3'
	Primer (reverse)	5'-TCA TAC CAG GGC TTG AGC TCA-3'
	Probe (antisense)	5'-AGA GCC CTT GCC CTA AGG ACA CCC CT-3'

**IL-6**	Primer (forward)	5'-CGAAAGTCAACTCCATCTGCC-3'
	Primer (reverse)	5'-GGCAACTGGCTGGAAGTCTCT-3'
	Probe (sense)	5'-TCAGGAACAGCTATGAAGTTTCTCTCCG-3'

**ICAM-1**	Primer (forward)	5'-AAACGGGAGATGAATGGTACCTAC-3'
	Primer (reverse)	5'-TGCACGTCCCTGGTGATACTC-3'
	Probe (sense)	5'-TGCCGTGCCTTTAGCTCCCGTG-3'

**rpL32**	Primer (forward)	5'-TGT CCT CTA AGA ACC GAA AAG CC-3'
	Primer (reverse)	5'-CGT TGG GAT TGG TGA CTC TGA-3'
	Probe (sense)	5'-TCG TAG AAA GAG CAG CAC AGC TGG CC-3'

### Fluorescent activated cell sorting (FACS) analysis

Peripheral blood cells were isolated from all groups after 24 hrs of reperfusion, washed and incubated with monoclonal antibodies for a cytotoxic T cell sub population (CD3^+ ^CD8^+ ^T cells). We used PE-mouse anti-rat CD8a and FITC-mouse anti-rat CD3 for this purpose. PE-mouse IgG1, K and FITC-mouse IgG3, K were used as the respective isotype negative controls. All antibodies and isotype controls were obtained from BD Pharmingen (San Jose, CA). FACS analysis was performed using "Cytomics FC 500 MPL" from Beckman Coulter (Brea, CA). Stained cells were expressed as a percentage of total leukocytes, after subtraction of their respective negative isotype controls.

### Western blot analysis

Tissue samples were first washed with ice-cold phosphate-buffered saline (PBS) and homogenized using T-PER buffer with protease and Halt-Phosphatase inhibitor cocktail (Pierce Chemical, Rockford, IL). The homogenates were centrifuged at 5000 rpm at 4^ο ^C for 10 min, to remove cell debris. All samples were assayed for protein concentration using a BCA assay (Pierce Chemical, Rockford, IL), after which the samples were stored at -80°C until use. Proteins were separated by SDS-polyacrylamide gel electrophoresis and transferred to a polyvinylidene difluoride membrane. The membrane was blocked with 5% bovine serum albumin (BSA) in PBS/Tween 20 solution. The blots were incubated with (Signal Transducer and activator of transcription) phospho-STAT-3 (pSTAT-3), STAT3 (Cell signaling Technology, Inc. Danvers, MA) and β-actin (Sigma-Aldrich St. Louis, MO) antibodies. After washing with PBS/Tween 20 solution, the blots were incubated with appropriate horseradish peroxidase- (HRP-) conjugated secondary antibodies, followed by development using an Enhanced Chemiluminescence detection kit (GE Healthcare, Chalfont St.Giles, Buckinghamshire, UK).

### Immunofluorescent staining

Rat brains were harvested after 3 or 24 hrs of reperfusion. They were then paraffin-embedded and sectioned. The sections were stained to determine the expression levels of Foxp3 and Bcl-xL, using methods previously described by Khan et al [15] with slight modifications. Briefly, sections were antigen-unmasked using 1 × citrate-based antigen unmask solution from Vector Labs (Burlingame, CA) using the manufacturer's protocol. After antigen unmask, the slides were blocked with 10% goat normal serum and permeabilized with 0.01% triton X100 in PBS, followed by primary antibody incubation with either FITC conjugated Foxp3 (eBioscience, San Diego, CA) (1:100 dilution) or rabbit anti-Bcl-xL (Cell Signaling Technology, Inc. Danvers, MA) (1:100 dilution). Bcl-xL primary antibody-treated slides were then washed (with 0.01% tween20 in PBS) and incubated with secondary antibody (goat anti-rabbit IgG-FITC) (1:300 dilution). All slides were then washed and mounted with vector shield mounting media with DAPI from Vector Labs (Burlingame, CA). The slides were observed with an Olympus 1X81 microscope and images were processed using Adobe Photoshop software (San Jose, CA). Using image J analysis software http://rsbweb.nih.gov/ij/, all images were converted into binary images then Foxp3/Bcl-xL-positive cells were counted and analyzed. Areas were randomly selected from 3-5 sections (for each animal) to obtain counts of positively stained cells, which were then normalized to the sham group and plotted as fold change.

### TUNEL staining

Terminal deoxynucleotidyl transferase dUTP nick end labeling (TUNEL) was performed using a kit from Chemicon (Billerica, MA). The manufacturer's instructions were followed for processing and staining of sections. Finally, all slides were then washed and mounted with vector shield mounting media with DAPI from Vector Labs (Burlingame, CA). The slides were observed with an Olympus 1X81 microscope and images were processed using Adobe Photoshop software (San Jose, CA). TUNEL-positive cells were counted using Image J analysis software http://rsbweb.nih.gov/ij/, as described above. Areas in and around the penumbra were randomly selected from 3-5 sections (for each animal) to obtain counts of positively stained cells, which were then normalized to sham group and plotted as fold change.

### Statistics

Graphpad Prism 5.0 version (GraphPad software, San Diego CA, USA) was used to perform statistical calculations. Data were compared using one way ANOVA followed by Bonferroni post-hoc analysis. A '*p*' value of less than 0.05 was considered significant, all values were represented as mean ± SD for *n *determination.

## Results

### MPO activity assay

**Figure 1:**
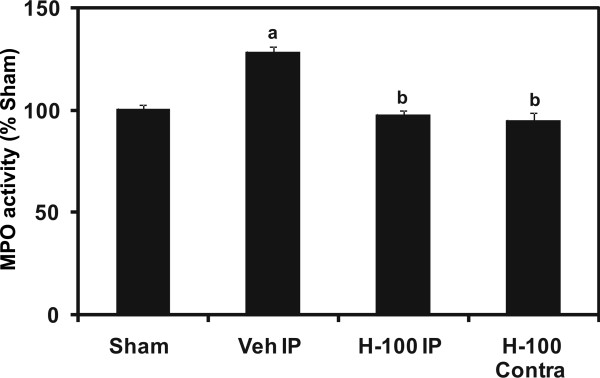
**Myeloperoxidase (MPO) activity assay in cortical (ipsilateral) brain after 24 hrs of reperfusion**. The graph is a representation of *n *= 5 experiments and results are expressed as % of Sham. "a" for *p *≤ 0.01 when compared to sham. "b" for *p *≤ 0.01 when compared to vehicle group, "c" for *p *≤ 0.01 when compared to H-100 IP group and ,"d" for *p *≤ 0.01 when compared to H-100 Contra group.

Myeloperoxidase (MPO) is the most abundant enzyme in neutrophils, it is also present in monocytes, and is considered to be a primary focus of inflammatory pathologies. MPO assays performed on ipsilateral cortical brain samples after 24 hrs of I/R showed a significant increase in activity in vehicle-treated rats versus sham operated animals. Hawthorn extract treatment significantly (*p *≤ 0.01) decreased this activity (Figure 1), suggesting that Hawthorn-pretreated cortical brain tissue had decreased exposure to potential tissue damaging cells such as neutrophils or other inflammatory cells.

### IL-10 levels in brain

**Figure 2:**
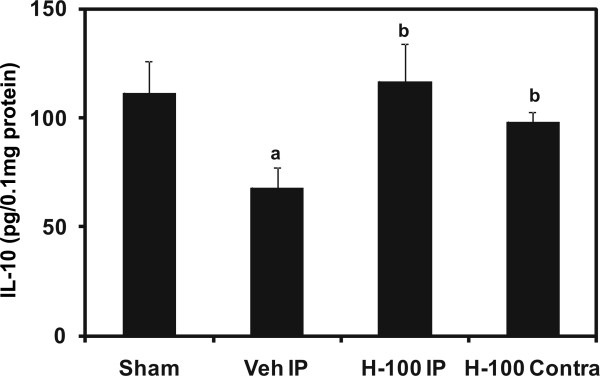
**Interleukin-10 levels in ipsilateral brain tissue after 24 hrs of reperfusion**. Data represented as mean ± SD (*n *= 6). IL-10 levels are expressed as picograms/0.1 mg of total protein. "a" for *p *≤ 0.01 when compared to sham. "b" for *p *≤ 0.01 when compared to vehicle group, "c" for *p *≤ 0.01 when compared to H-100 IP group and ,"d" for *p *≤ 0.01 when compared to H-100 Contra group.

IL-10 is an immuno-suppressant that is mainly secreted by regulatory T cells. We found IL-10 levels to be significantly (n = 6 per group; *p *≤ 0.01) lower in ipsilateral brain after 24 hrs of reperfusion in the vehicle group, compared to sham. However, Hawthorn extract was able to maintain IL-10 expression close to levels observed in sham (Figure 2), possibly by enhancing the presence or activity of regulatory T cells in the brain. The contralateral IL-10 levels in vehicle and Hawthorn-pretreated groups were found to be similar.

### RT-PCR of proinflammatory mediators

**Figure 3:**
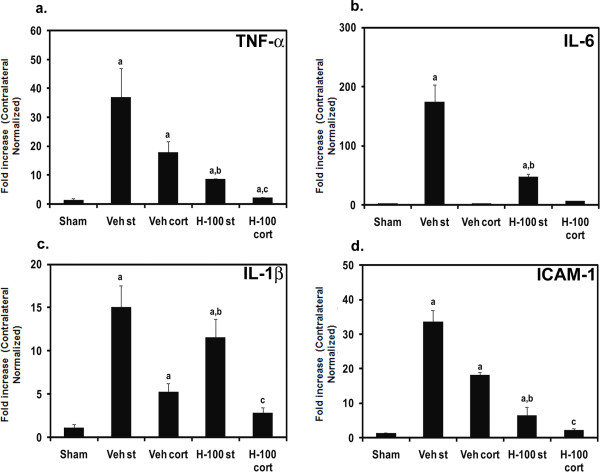
**Proinflammatory cytokine and ICAM-1 expression levels in striatum/cortex of the ipsilateral brain after ischemia followed by 3 hrs of reperfusion**. Bar graphs (a) TNF-α, (b) IL-6, (c) IL-1β and (d) ICAM-1 indicate expression levels (fold increases) as mean ± SD (*n *≥ 6) for two different experiments. Data represented as fold increase normalized to respective contralateral side; "a" for *p *≤ 0.05 when sham compared to other groups; "b" for *p *≤ 0.05 when vehicle striatum (st) compared with H-100 striatum (st) and "c" for *p *≤ 0.05 when vehicle cortex (cort) compared to H-100 cortex (cort).

TNF-α, IL-1β, IL-6 and ICAM-1 gene expressions were studied in different regions of brain (striatum and cortex) after 3 hrs of reperfusion. The vehicle control group showed a significant fold increase (n ≥ 6 per group; *p *≤ 0.01) in the above mentioned genes in the striatum of the ipsilateral hemisphere when compared to Hawthorn-treated group. We also observed a significant reduction in the induction of both TNF-α and IL-1β mRNA in the cortex of treated brain (8.7 and 1.9 fold, respectively). However we did not observe a significant difference between vehicle and treated groups for expression of the IL-6 gene, in the cortex region (Figure 3).

### FACS analysis for cytotoxic T cells

**Figure 4:**
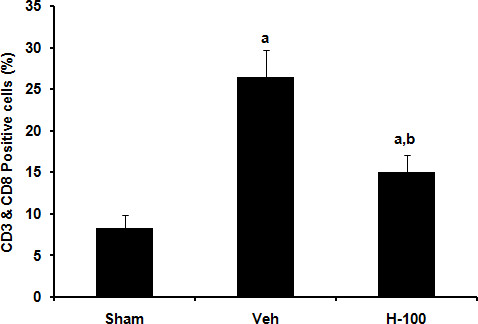
**Fluorescence assorted cell sorting analysis of cytotoxic T cells (CD3^**+ **^**CD8**^**+**^)**. Data presented as mean ± SD (*n *≥ 4) CD3- & CD8-positive cells as a percentage of total leukocytes; "a" for *p *≤ 0.05 when compared to sham group; "b" for *p *≤ 0.05 when compared to vehicle group.

The circulating cytotoxic T cell subpopulation (CD3^+ ^CD8^+ ^cells) is a good indicator of systemic immune response to I/R injury. The cells could potentially enter the brain through a leaking blood-brain barrier or even through blood vessels with the help of adhesion molecules. Peripheral blood mononuclear cells (PBMCs) were stained for this cytotoxic T cell sub-population. In our hands the percentage of cytotoxic T cells was found to be significantly increased in vehicle group when compared to sham group (n ≥ 4 per group; *p *≤ 0.01). Hawthorn pretreatment significantly reduced these levels (Figure 4).

### Hawthorn pretreatment increases STAT-3 phosphorylation in rat brain after I/R

**Figure 5:**
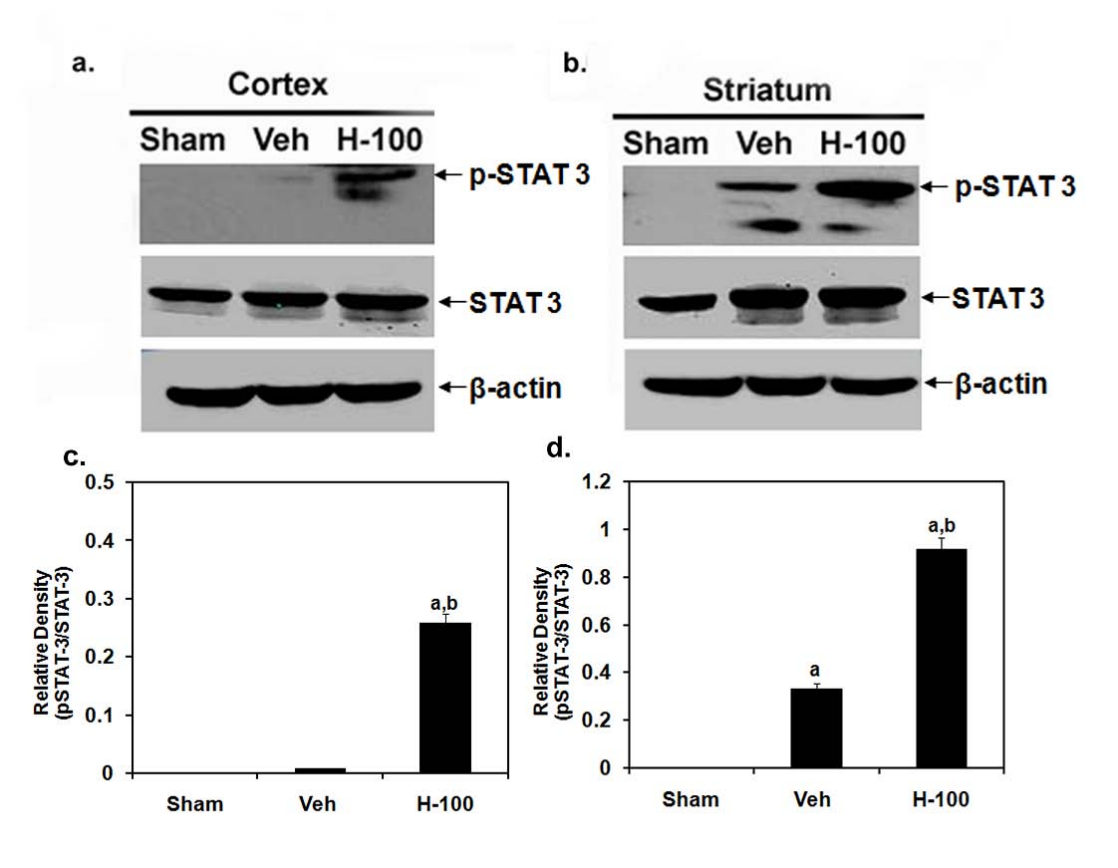
**Western blot analysis of pSTAT-3/STAT-3 expression in tissue after 24 hrs of reperfusion**. Representative blot image of pSTAT-3/STAT-3 in cortex (a) and striatum (b). The last panel shows bands for β-actin in the respective samples. Also shown are graphical presentation of results indicated as relative densities (pSTAT-3/STAT-3) for cortex (c) and striatum (d). Relative densities are presented as mean ± SD (*n *= 4); "a" for *p *≤ 0.01 when compared to sham group; "b" for *p *≤ 0.01 when compared to vehicle group.

After 24 hrs of reperfusion there was an increase in STAT-3 phosphorylation in Hawthorn-pretreated brain. Though the levels in vehicle ipsilateral hemisphere were higher than sham group, they were significantly less when compared to levels found in Hawthorn-pretreated brain. This was observed in both cortex and striatum regions of brain. However, total endogenous STAT-3 expression was unchanged (Figure 5).

### Immunofluorescent staining in brain sections

**Figure 6:**
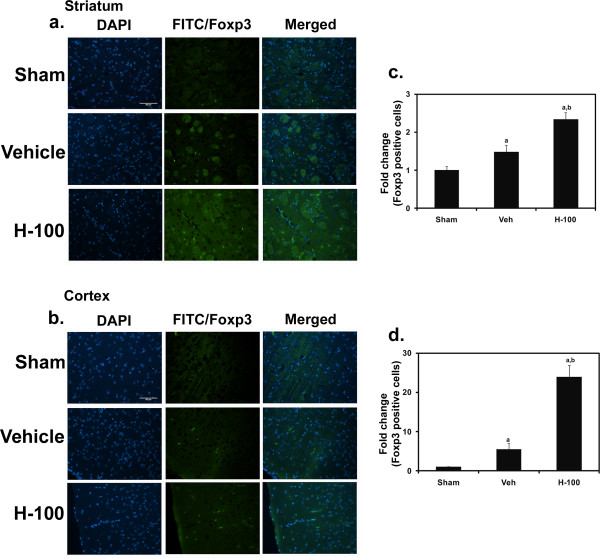
**Foxp3 immunofluorescent staining in brain after 3 hrs of reperfusion**. Photographs show Foxp3 staining in striatum (a) and cortex (b) regions of brain in all three groups (Sham, Vehicle and H-100). Green staining represents Foxp3-positive cells, and DAPI (which stains the nucleus) is in blue. A merged image of DAPI and Foxp3 to indicate cell-specific staining for each group is also shown. Images were taken at 200 × magnification. Scale bar = 80 μm. Graphical representation of Foxp3-positive cell counts in striatum (c) and cortex (d) regions, indicated as sham-normalized fold change are also shown; "a" for *p *≤ 0.05 when compared to sham group; "b" for *p *≤ 0.05 when compared to vehicle group.

**Figure 7:**
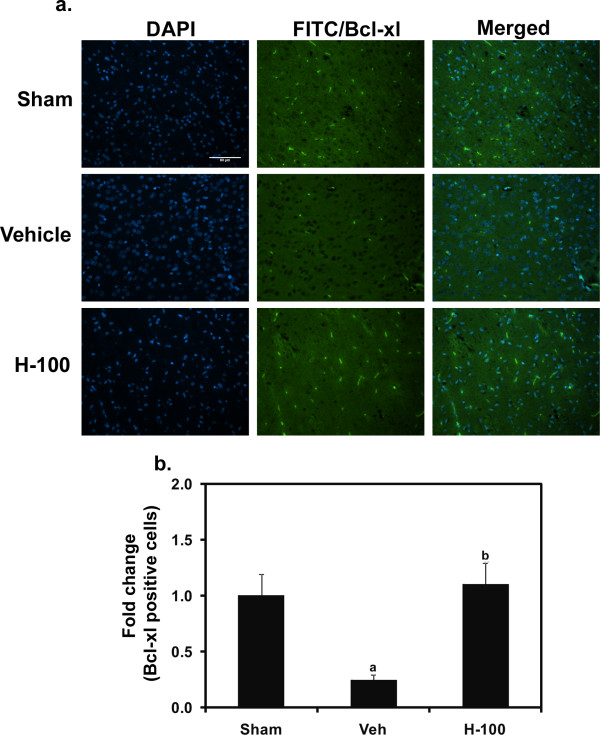
**Immunofluorescent staining for Bcl-xL in and around the penumbra of ipsilateral brain after 24 hrs of reperfusion**. Representative images of Bcl-xL-positive cells (FITC - green fluorescence) along with nuclear staining (DAPI - blue) in Sham, Vehicle and H-100 groups. A merged image of DAPI and Bcl-xL to indicate cell specific staining for each group is also shown. Images were taken at 200 × magnification. Scale bar = 80 μm. A graphical representation of Bcl-xL-positive cell counts, indicated as sham-normalized fold change is also shown; "a" for *p *≤ 0.05 when compared to sham group; "b" for *p *≤ 0.05 when compared to vehicle group.

Vehicle control rat brain sections obtained after 3 hrs of reperfusion showed very few Foxp3-positive cells (stained in green) in the ipsilateral hemisphere (striatum and cortex). However, we found a larger number of cells to be Foxp3-positive in the same regions of Hawthorn-pretreated rats (Figure 6). Bcl-xL staining was performed in brain sections after 24 hrs of reperfusion (Figure 7), and showed an increase in the number of Bcl-xL-positive cells (stained in green) in the H-100 group compared to the vehicle control group. Images were captured in regions that appeared to have equal numbers of brain cells (indicated by their nuclei, stained in blue) across different groups. All photographic images were taken at a 200 × magnification.

### TUNEL assay in brain tissue

**Figure 8:**
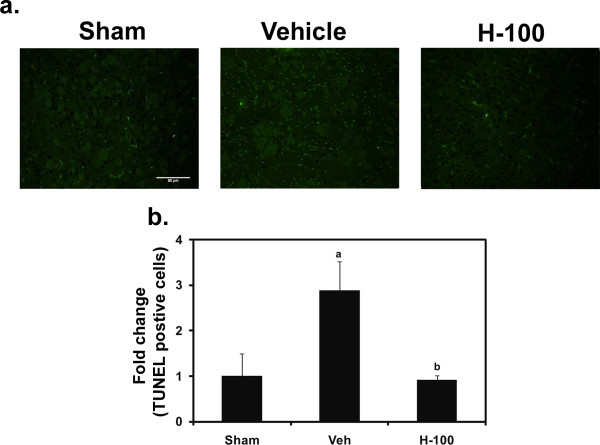
**TUNEL staining representing apoptotic cells in and around the penumbra of ipsilateral brain after 24 hrs of reperfusion in all three groups (Sham, Vehicle and H-100)**. The green staining represents TUNEL-positive cells. Images were taken at 100 × magnification. Scale bar = 80 μm. A graphical representation of TUNEL-positive cell counts, indicated as sham-normalized fold change is also shown; "a" for *p *≤ 0.05 when compared to sham group; "b" for *p *≤ 0.05 when compared to vehicle group.

Programmed cell death plays a pivotal role in the loss of brain cells during I/R. We used TUNEL staining to look for apoptotic cells in and around the penumbra of the ipsilateral hemisphere. After 24 hrs of reperfusion, sections from vehicle-treated rat brain, showed a significant increase in TUNEL-positive cells in the penumbra of ipsilateral hemisphere, compared to sham brain tissue. Hawthorn pretreatment was able to reduce TUNEL-positive cells following I/R-induced injury (Figure 8).

## Discussion

Activation of an inflammatory response is considered to be a key event associated with most human pathologies like stroke and atherosclerosis [16,17]. Alleviating the immune response has been found to be favourable in the prognosis of such disorders [18]. Furthermore, Fu et al [19] showed an increase in proinflammatory cytokine expression in lumbosacral spinal cord and urinary bladder of rats that were subjected to transient and permanent MCAO, two sites far from the primary point of infarct, suggesting neural death may influence spinal cord and urinary bladder dysfunction in clinical stroke. Apart from this, CNS injury can lead to secondary immunodeficiencies resulting in infections [20]. With several immune system-related factors (cytokines, leukocytes, etc) known to affect the brain and other organs during ischemia/reperfusion injury, a clear understanding of the mechanisms and functions of these factors could be key for developing beneficial therapeutic strategies. A successful therapeutic strategy would include suppression of proinflammatory cytokines, decreased autoimmunity to CNS antigens, increases in anti-inflammatory cytokines, reduction in the recruitment of inflammatory cells, etc. Previously, our lab established the neuroprotective effect of Hawthorn extract in a rat stroke model [2]. Here, we studied different immune responses occurring during ischemia/reperfusion in vehicle- and Hawthorn extract-pretreated brain. We used the MCAO-induced cerebral ischemia/reperfusion injury model for this study. The degree of injury was consistent with an occlusion period of 75 minutes and, by using commercially available sutures for occluding the MCA, we had a 26% mortality rate with this model.

Animal models of cerebral stroke indicate that interfering with the inflammatory response that occurs during brain I/R injury may be beneficial [21,22]. Preclinical evidence also demonstrates that immune system tolerance to central nervous system (CNS) antigens improves outcome from stroke [23,24]. Injury to the CNS disturbs the well balanced interplay between the immune system and the CNS [25], which results in further tissue damage through aberrant activation of the immune system. Studies have shown that ischemia/reperfusion injury induces expression of cell adhesion molecules and cytokines in the brain through activation of the nuclear transcription factor, NF-κB [14]. It is well established that NF-κB is activated during MCAO-induced I/R injury. Reducing its activity is associated with reduction in infarct volume after MCAO [26]. NF-κB is mainly activated by TNF-α. 6-O-acetyl shanzhiside methyl ester has been found to mediate its neuroprotective effect in the MCAO model by blocking TNF-α-induced activation of NF-κB [27]. In our model, proinflammatory cytokines (TNF-α, IL-6 and IL-1β) were induced after 3 hrs of reperfusion in different regions of the ipsilateral vehicle-treated brain, and Hawthorn extract significantly decreased their induction by at least 1.3-fold in striatum. This may have resulted in decreased NF-κB activation thereby reducing further damage to the brain. Microglial cells are the main source of TNF-α and IL-1β. A significant reduction in TNF-α and IL-1β expression in the Hawthorn extract-treated group may indicate a suppression of resident microglial cell activation by the extract.

Foxp3-positive regulatory T Cells (T_regs_) are important for maintaining peripheral tolerance, preventing autoimmune diseases and limiting chronic inflammatory diseases [28,29]. One of the mechanisms by which T_regs _mediate their suppressive effect is by secreting inhibitory cytokines such as IL-10. IL-10 is well known for its positive effects in cerebral ischemia in rats [30]. Hawthorn pretreatment was able to significantly increase IL-10 protein expression in ipsilateral brain by at least 30% when compared to vehicle-treated rats after 24 hrs of reperfusion. Furthermore, Foxp3 (marker for T_regs_) staining in brain sections from animals with 3 hr of reperfusion complemented the IL-10 results, in that we observed increased Foxp3 staining in H-100-treated ipsilateral brain when compared to vehicle-treated tissue. The abundance of T_regs _in Hawthorn-treated brain following I/R may have influenced the increased expression of IL-10. In addition, T_regs _can be induced, adapted or converted from effector T cells during inflammatory processes in peripheral tissues for therapeutic purposes [31-33], especially the IL-10-secreting T regulatory type 1 (T_r_1), defined by their specific cytokine production profile (both IL-10 and transforming growth factor-β). These cells also have the ability to suppress antigen-specific effector T cell responses via their cytokine-dependent mechanisms. However, T_r_1 cells do not express Foxp3. The significantly elevated levels of IL-10 in Hawthorn-pretreated brain in our model indicates the possible involvement of T_r_1 cells in addition to T_regs_. These cells are also capable of antagonizing the production of proinflammatory cytokines (TNF-α and IFNγ), that induce secondary brain damage during stroke [34]. Therefore, Hawthorn extract not only improved anti-inflammatory response but also suppressed the proinflammatory immune response and secondary brain damage. Our results indicate that T_regs _may have a critical role in this immunomodulatory response.

Myeloperoxidase, abundantly present in neutrophils, produces hypochlorous acid from hydrogen peroxide and chloride ion during the neutrophils' respiratory burst (abundance of reactive oxygen species). Hypochlorous acid is highly cytotoxic and has been demonstrated to damage CNS tissue during inflammation [35]. Further, myeloperoxidase activity assay has been successfully used to confirm inflammatory cell activation and recruitment in brain after MCAO [13]. We performed this assay to establish a relationship between the neuroprotective effect of Hawthorn extract and its immunomodulatory properties in MCAO-induced brain injury (cortex) after 24 hrs of reperfusion. As expected, there was a significant increase (28%) in MPO activity in vehicle brain (ipsilateral cortex) when compared to sham group. Hawthorn pretreatment was able to reduce this activity to the levels observed in sham (Figure 1). This indicates that there was a significant suppression of neutrophil activation and recruitment leading to less tissue damage.

Intracellular adhesion molecule-1 (ICAM-1) is essential for the recruitment and trafficking of leukocytes through vessels and in T cell/macrophage interactions [36]. A recent review describes how adhesion molecules in general have effects on infarct size in the MCAO model [37]. Another group has shown that the neuroprotective effect of the antioxidant caffeic acid phenyl ester is partly due to its ability to minimize the expression of I/R-induced ICAM-1 [15]. In our study, the observed increase in ICAM-1 gene expression in ipsilateral brain after 3 hrs of reperfusion in vehicle treated group was decreased with H-100 pretreatment. This may have minimized leukocyte trafficking to the brain.

To better understand the neuroprotective mechanism offered by Hawthorn extract we looked at STAT-3 phosphorylation after 24 hrs of reperfusion in ipsilateral brain. H-100-pretreated rats showed significantly higher levels of phosphorylated STAT-3 when compared to vehicle-treated rats, while in the sham group no phosphorylation was detected. H-100 pretreatment did not have any effect on the basal expression of STAT-3; all 3 groups had similar STAT-3 levels after 24 hrs of reperfusion. Dziennis et al [38] has shown pSTAT-3 to be a positive modulator in the neuroprotective function offered by estradiol in a similar stroke model. They claim that, in the estradiol-treated group, STAT-3 is phosphorylated and translocates into the nucleus to promote transcription of the Bcl-2 family of proteins. Thus, we probed for Bcl-xL, one of the anti-apoptotic members of Bcl-2 family proteins to see if this was the case in our model. As seen in figure 7, immunofluorescent staining performed in brain sections from animals after 24 hr of reperfusion showed that, in comparable regions, H-100-treated brain expressed higher levels of Bcl-xL than vehicle-treated brain. We further performed TUNEL staining of brain tissue to see if higher Bcl-xL expression in the treated group translated to an anti-apoptotic effect. As expected, there was a significant increase in TUNEL-positive cells in ipsilateral vehicle-treated brain sections after 24 hrs of reperfusion, compared to the sham group, However, H-100 pretreatment was able to significantly decrease the number of TUNEL-positive cells to close to levels observed in the sham group (Figure 8). Apoptotic cell death can also occur by modulation of caspases. Granzyme-b, a serine protease found in the cytoplasmic granules of cytotoxic T cells, plays a vital role by activating several caspases and by initiating caspase-independent pathways that contribute to target cell death [39]. In the present study, the CD3^+ ^CD8^+ ^cytotoxic T cell population was significantly increased in the vehicle-treated group after I/R, However this cell population was reduced by at least 1.7-fold in the pretreated group (Figure 4), which may have reduced granzyme-b and caspase activation, thereby decreasing the cytotoxic T cell population in brain and contributing to reduced neuronal death due to apoptosis. This may explain the low level of TUNEL-positive cells seen in the pretreated group.

About 700,000 people suffer from stroke in America each year, and 200,000 of these are recurrent strokes [40]. A recent study has also shown that nearly two-thirds of stroke patients who are discharged after having an attack will die or be readmitted to the hospital within a year. This study analyzed ischemic stroke incidences from 625 hospitals from the US and reported the readmission rate as 62% within a year of the first stroke. Furthermore, patients who are in the high-risk category for stroke, such as those with hypertension, diabetes, dyslipidemia, etc; also form a significant proportion of the population [41]. These facts show the importance of controlling risk factors either through diet or treatment in this high risk group. Our present study shows that Hawthorn extract may be beneficial in patients prone to stroke by decreasing some of the immune system-mediated damage to the brain during stroke. Oxidative stress and an increase in ROS have been hallmark features associated with stroke, and Hawthorn extract pretreatment has been shown to decrease oxidative stress and associated damage [2]. Adding to this, Hawthorn extract has already been tested in humans. It has been extensively used and well advocated as an oral treatment option for chronic heart failure. According to the New York Heart Association, the German Commission E has approved the use of Hawthorn extract (leaf and flower) by patients suffering from heart failure graded stage II [42]. A review of 14 double-blind, placebo-controlled, randomised clinical trials (RCTs) found that, in addition to conventional drugs, oral treatment of Hawthorn extract could be an option for chronic heart failure [42]. The daily dose used in these clinical trials ranged between 160 mg and 1800 mg. Our results from the current and previous studies indicate that Hawthorn extract has beneficial anti-inflammatory and antiapoptotic effects in a cerebral stroke model, and this suggests that people who are prone to stroke or who have suffered their first stroke may potentially benefit from an oral pretreatment schedule with Hawthorn extract. Further clinical studies are essential to determine the clinical impact and exact dose.

## Conclusions

In summary, we observed decreased neutrophil and CD3^+^CD8^+ ^cytotoxic T cell activity in Hawthorn extract-pretreated animals following I/R. There was also a significant down-regulation of pro-inflammatory mediators (TNF-α, IL-6, IL-1β and ICAM-1) in brain. We found that Hawthorn pretreatment helped increase the regulatory T cell population in brain following ischemia/reperfusion injury. These cells mediated their immunosuppressive role with the help of IL-10, which was also found to be elevated in pretreated brain. Phosphorylation of STAT-3 in brain of the Hawthorn-treated group was elevated, which may have helped to minimize apoptosis in brain during reperfusion. Taken together, our results suggest that Hawthorn extract has an immunomodulatory effect that plays a crucial role in the observed neuroprotection after MCAO-induced stroke. However, further studies are needed to clarify the mechanism of this immunomodulatory effect of Hawthorn extract.

## Competing interests

The authors declare that they have no competing interests.

## Authors' contributions

CE was involved in designing, planning and execution of all experiments mentioned in this manuscript, he was also involved in drafting the manuscript. ND participated in drafting manuscript and revising it to capture vital intellectual content and provided final approval for publication. All authors have read and approved the final version of the manuscript.
